# Acute nitric oxide synthase inhibition induces greater increases in blood pressure in female versus male Wistar Kyoto rats

**DOI:** 10.14814/phy2.15771

**Published:** 2023-08-07

**Authors:** Ahmed A. Elmarakby, Karim M. Saad, G. Ryan Crislip, Jennifer C. Sullivan

**Affiliations:** ^1^ Departments of Oral Biology & Diagnostic Sciences Augusta University Augusta Georgia USA; ^2^ Department of Pharmacology and Toxicology, Faculty of Pharmacy Mansoura University Mansoura Egypt; ^3^ Departments of Physiology Augusta University Augusta Georgia USA

**Keywords:** blood pressure, L‐NAME, renal hemodynamics, sex, VNIO, WKY

## Abstract

Nitric oxide (NO) contributes to blood pressure (BP) regulation via its vasodilatory and anti‐inflammatory properties. We and others previously reported sex differences in BP in normotensive and hypertensive rat models where females have lower BP than age‐matched males. As females are known to have greater NO bioavailability than age‐matched males, the current study was designed to test the hypothesis that anesthetized female normotensive Wistar Kyoto rats (WKY) are more responsive to acute NOS inhibition‐induced increases in BP compared to male WKY. Twelve‐week‐old male and female WKY were randomized to infusion of the nonspecific NOS inhibitor N^G^‐nitro‐L‐arginine methyl ester (L‐NAME, 1 mg/kg/min) or selective NOS1 inhibition with vinyl‐L‐NIO (VNIO, 0.5 mg/kg/min) for 60 min. Mean arterial BP, glomerular filtration rate (GFR), urine volume, and electrolyte excretion were assessed before, and during L‐NAME or VNIO infusion. L‐NAME and VNIO significantly increased BP in both sexes; however, the increase in BP with L‐NAME infusion was greater in females versus males compared to baseline BP values. Acute infusion of neither L‐NAME nor VNIO for 60 min altered GFR in either sex. However, urine volume, sodium, chloride and potassium excretion levels increased comparably in male and female WKY with L‐NAME and VNIO infusion. Our findings suggest sex differences in BP responses to acute non‐isoform‐specific NOS inhibition in WKY, with females being more responsive to L‐NAME‐induced elevations in BP relative to male WKY. However, sex differences in the BP response did not coincide with sex differences in renal hemodynamic responses to acute NOS inhibition.

## 1 INTRODUCTION

Nitric oxide (NO) plays a crucial role in blood pressure (BP) regulation via its vasodilatory, natriuretic, and anti‐inflammatory properties (Brinson et al., 2013; Herrera & Garvin, 2005; Layton & Sullivan, 2019). NO is produced via the conversion of the amino acid L‐arginine to L‐citrulline by the enzyme nitric oxide synthase (NOS) (Stuehr, 2004). Three main NOS isoforms have been identified based on distinct cellular distribution and regulation; neuronal NOS (NOS1), inducible NOS (NOS2), and endothelial NOS (NOS3) (Sullivan et al., 2010; Villanueva & Giulivi, 2010). Loss of NOS‐mediated vascular control or decreased NO availability by increasing superoxide production contributes to the development and progression of hypertension (Li et al., 2015). Importantly, both NOS1 and NOS3 have been shown to play a key role in BP regulation (Sasser et al., 2015; Sullivan et al., 2010). More specifically, the kidney is known to play a crucial role in BP regulation and NO has been established to play a pivotal role in this process (Hyndman et al., 2013; Loria et al., 2014; Sasser et al., 2015). For example, the renal inner medullary collecting duct expresses both NOS1 and NOS3 and has the highest NOS activity in the kidney (Hyndman et al., 2013; Sasser et al., 2015). Moreover, medullary infusion of NOS1 inhibitors in male rats fed high salt diet increases BP (Mattson & Bellehumeur, 1996), confirming the importance of NOS1 in BP regulation.

Previous studies have demonstrated sex differences in BP with premenopausal females having a lower BP than age‐matched males (Hilliard et al., 2013; Sabolic et al., 2007; Sandberg & Ji, 2012; Wiinberg et al., 1995). Experimentally, sexual dimorphisms in BP regulation are also evident in normotensive rats (Alhashim et al., 2022; Sandberg & Ji, 2012). For example, female Wistar Kyoto rats (WKY) have significantly lower BP compared to age‐matched males (Alhashim et al., 2022). Although the molecular mechanism(s) by which females maintain a lower BP versus males remains unclear, greater expression and activation of the NO/NOS system in females has been implicated. Studies have shown that lower BP in females coincides with a greater NO bioavailability compared to males, which can be attributed to either greater NO production or decreased rate of NO scavenging by superoxide since females had also lower oxidative stress than males (del Campo et al., 2009; Ide et al., 2002; Kahonen et al., 1998; McIntyre et al., 1997; Reckelhoff et al., 1998). Although female rats have greater NO compared to males (Glushkovskaya‐Semyachkina et al., 2006; Loria et al., 2014), there are conflicting data in the literature regarding the impact of sex on the response to chronic NOS inhibition in normotensive rats where normotensive female rats have shown to be more sensitive (Wang et al., 2003), less sensitive (Sainz et al., 2004), or equally sensitive (Wu et al., 2001) to NOS inhibition‐induced hypertension compared to age‐matched males. As hypertension is often associated with renal hemodynamic changes (Reckelhoff et al., 1998), the current study was designed to test the hypothesis that female WKY rats will have higher sensitivity to acute NOS inhibition than males. We postulate that NOS inhibition‐induced increases in BP and renal hemodynamic changes will be greater in female versus male WKY.

## 2 METHODS

Twelve‐week‐old male and female WKY were used in this study (Envigo, Indianapolis, IN). All experiments were conducted in accordance with the *National Institutes of Health Guide for the Care and Use of Laboratory Animals* and approved and monitored by the Augusta University Institutional Animal Care and Use Committee. Rats were maintained at the Augusta University animal care facility for 1 week before the study for acclimation purposes. Rats were allowed free access to food and water and were housed in temperature‐controlled conditions with a 12‐h light–dark cycle.

Previously published studies have shown that acute NOS inhibition with L‐NAME infusion at 200 μg/kg/min increased BP in male Wistar rats (Nafrialdi et al., 1994). Acute L‐NAME infusion also significantly increased MAP at 50 μg/kg/min (Dobrowolski et al., 2015). For NOS 1 inhibition with VNIO, Shi et al. previously found that l‐VNIO infused to achieve a concentration of 1 μmoL/L in renal arterial blood augmented myogenic autoregulation in male Wistar rats, yet this concentration did not cause significant vasoconstriction (Shi et al., 2006). We could not find additional studies in the literature evaluating the effect of NOS1 inhibition with VNIO on BP acutely. Thus, initial studies in male and female WKY (*n* = 4–5) were performed to determine BP responses to graded bolus doses of the nonspecific NOS inhibitor L‐NAME (0.08–2.5 mg/kg iv; Sigma, St. Louis, MO) or the NOS1‐specific inhibitor VNIO (0.32–10.0 mg/kg iv; Cayman Chemical, Ann Arbor, MI).

A separate set of male and female WKY (*n* = 5–6/group) was then used to determine the effects of acute NOS inhibition with L‐NAME or VNIO on BP, glomerular filtration rate (GFR), electrolyte excretion, hematocrit, and urine flow rate as previously described (Elmarakby et al., 2016). Briefly, rats were anesthetized with Inactin (100 mg/kg ip, Sigma, St. Louis, MO) and placed on a servo‐controlled heating table to maintain rectal temperature constant at 37°C. A tracheostomy was performed to prevent obstructed breathing using polyethylene (PE)‐200 tubing. Using a PowerLab data acquisition system (AD Instruments Inc., Colorado Springs, CO), mean arterial BP was recorded by a PE catheter (PE‐50) inserted into the carotid artery. The jugular and femoral veins were cannulated using PE‐50 catheters for continuous infusion of 6.2% BSA in PBS at a rate of 1.8 mL/h or fluorescein isothiocyanate‐inulin (Sigma, 0.5 mg/kg/min), respectively. Another PE catheter (PE‐90) was placed into the urinary bladder for urine collection. After a 60‐min equilibration period, a 30‐min baseline urine collection period was begun that included a blood sample taken at the midpoint of the period to measure hematocrit values. Following the first 30‐min baseline urine collection, rats were randomized to treatment with L‐NAME infusion (1 mg/kg/min) or VNIO infusion (0.5 mg/kg/min) for 60 min. Two additional 30‐min urine collection periods were obtained during L‐NAME or VNIO infusion with a blood sample taken at the midpoint for measurement of inulin concentration. Urine volume was measured gravimetrically. Plasma and urinary inulin concentrations were determined using a fluorospectrometer and values were used to calculate inulin clearance as an indicator of GFR based on the following formula: GFR = (urine flow × urine [inulin])/plasma [inulin]. Urinary potassium and chloride concentrations were determined by ion selective electrodes (Medica Easylyte, Bedford, MA). Urinary sodium concentration was measured using Analyst 200 Atomic Absorption Spectrometer (Perkin Elmer, Waltham, MA).

### 2.1 Statistical analysis

Data are presented as mean ± SEM. ANOVA for repeated measurements was used to compare BP changes in response to graded bolus doses of L‐NAME or VNIO in male and female WKY in addition to the comparison of BP response to each bolus dose of NOS inhibitors in male and female WKY to their corresponding baseline BP values using unpaired‐*t*‐test. Assessment of BP and renal hemodynamic changes to L‐NAME or VNIO infusion in male and female WKY were compared using two‐way ANOVA followed by Tukey's post hoc test, Factor 1 was sex and Factor 2 was L‐NAME or VNIO treatment. Differences were considered statistically significant with *p* < 0.05. Analyses were performed using GraphPad Prism version 8.0 software (GraphPad Software Inc.)

## 3 RESULTS

### 3.1 NOS inhibition results in dose‐dependent increases in BP in male and female WKY

**FIGURE 1:**
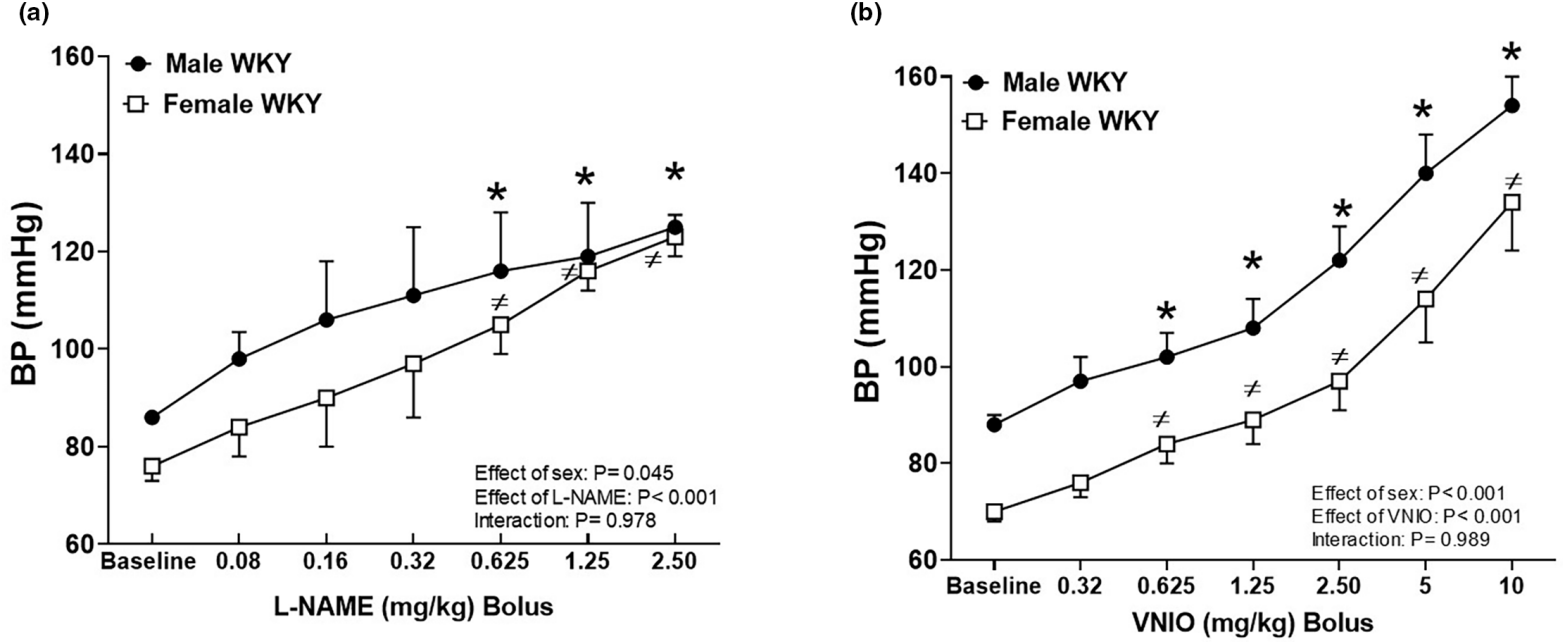
NOS inhibition results in dose dependent increases in BP in male and female WKY. BP responses to graded bolus doses of the nonspecific NOS inhibitor L‐NAME (0.08–2.5 mg/kg, i.v) (a) or the specific NOS1 inhibitor VNIO (0.32–10.0 mg/kg, iv) (b) in male and female WKY. Data are expressed as mean ± SEM; *n* = 4–5 in each group. *Indicates *p* < 0.05 versus corresponding male WKY baseline and ^≠^ indicates *p* < 0.05 versus corresponding female WKY baseline.

Initial studies were performed to determine BP responses to graded bolus doses of the nonspecific NOS inhibitor L‐NAME or the specific NOS1 inhibitor VNIO in male and female WKY. Male WKY had a greater BP than females at baseline (Figure 1a,b). Bolus injections of graded doses of L‐NAME (0.08–2.5 mg/kg, iv; Figure 1a) produced a dose‐dependent increase in BP in both male and female WKY (effect of sex, *p* = 0.045; effect of L‐NAME, *p* < 0.001; interaction, *p* = 0.978). Sex differences in BP were abolished following injection of 1.25 mg/kg or higher dose of L‐NAME (Figure 1a). Bolus injections of graded doses of VNIO (0.625/kg to 10 mg/kg, iv; Figure 1b) also produced a dose‐dependent increase in BP in both male and female WKY. Sex differences in BP were maintained throughout all doses of VNIO (effect of sex, *p* < 0.001; effect of VNIO, *p* < 0.001; interaction, *p* = 0.989).

### 3.2 Female WKY are more sensitive to L‐NAME‐induced increases in BP versus males

**FIGURE 2:**
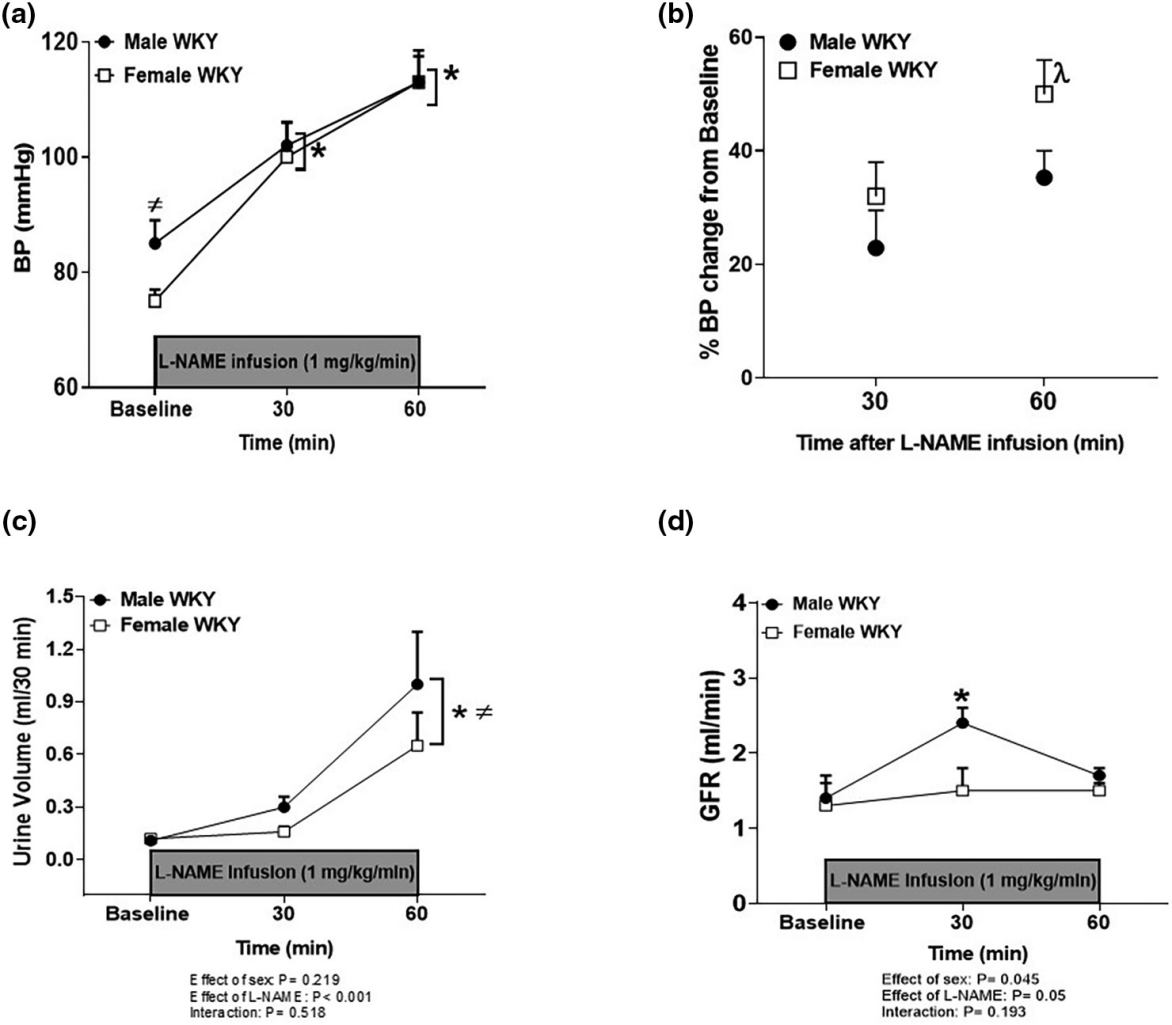
Female WKY are more sensitive to L‐NAME induced increases in BP versus males. BP prior to and during L‐NAME infusion (1.0 mg/kg/min, i.v) for 60 min in anesthetized male and female WKY (a). All data are expressed as mean ± SEM; *n* = 5–6 in each group * indicates *p* < 0.05 versus baseline values of either male or female WKY and ^≠^ indicates *p* < 0.05 versus baseline female WKY. Percent change in BP from baseline values in male and female WKY after 30‐min and 60‐min infusion of L‐NAME (b). ^λ^ Indicates *p* < 0.05 versus male WKY after 30 min of L‐NAME infusion. Urine volume (c) and glomerular filtration rate (GFR, d) prior to and during acute L‐NAME infusion (1.0 mg/kg/min, i.v) for 60 min in anesthetized male and female WKY. *Indicates *p* < 0.05 versus baseline male or female WKY and ^≠^ indicates *p* < 0.05 versus 30 infusion of L‐NAME in male or female WKY.

Baseline BP was higher in male WKY versus females. We then infused a dose of L‐NAME (1 mg/kg/min, i.v) close to the dose that abolished the sex difference in the BP response to L‐NAME to determine if sex differences in BP response to NOS inhibition were also associated with sex differences in renal hemodynamic responses. Infusion of L‐NAME significantly increased BP in both male and female WKY (effect of L‐NAME, *p* < 0.001; interaction, *p* = 0.473). The sex difference in BP was lost after L‐NAME infusion due to a greater increase in BP in females (Figure 2a,b).

Infusion of L‐NAME for 60 min significantly increased urine volume in male and female WKY compared to baseline values (Figure 2c). There were no sex differences in urine volume in WKY at baseline or during L‐NAME infusion (effect of sex, p = 0.219; effect of L‐NAME, *p* < 0.001; interaction, *p* = 0.518). There were also no significant changes in hematocrit values in male or female WKY before or after L‐NAME infusion (Table 1, effect of sex, *p* = 0.139; effect of L‐NAME, *p* = 0.659; interaction, *p* = 0.980). Baseline GFR was similar in male and female WKY (Figure 2d). L‐NAME significantly increased GFR in male WKY following 30‐min of infusion compared to baseline, however GFR returned to baseline values by 60 min (Figure 2d). GFR was not altered by L‐NAME infusion in female WKY (effect of sex, *p* = 0.045; effect of L‐NAME, *p* = 0.05; interaction, *p* = 0.193). Baseline electrolyte excretion levels were comparable in male and female WKY (Table 1). Infusion of L‐NAME significantly increased urinary sodium, chloride and potassium excretion levels compared to baseline values in both male and female WKY with no sex differences in these parameters (Table 1).

**TABLE 1:** L‐NAME increased urinary sodium, chloride and potassium excretion levels compared to baseline values without changing hematocrit values.

Parameter	Baseline		30 min after L‐NAME infusion		60 min after L‐NAME infusion	
Hematocrit (%)	Male	Female	Male	Female	Male	Female
	42.3 ± 1.0	41.0 ± 1.0	43.3 ± 1.1	41.6 ± 1.3	43.3 ± 1.3	42.0 ± 1.1
Na+ Excretion (μmol/min)	Male	Female	Male	Female	Male	Female
	0.2 ± 0.03	0.1 ± 0.02	0.7 ± 0.2	0.3 ± 0.1	2.9 ± 0.8*	3.5 ± 1.0*
Cl− Excretion (μmol/min)	Male	Female	Male	Female	Male	Female
	0.6 ± 0.2	0.4 ± 0.1	1.6 ± 0.4	1.0 ± 0.3	4.6 ± 1.5*	4.6 ± 1.4*
K+ Excretion (μmol/min)	Male	Female	Male	Female	Male	Female
	1.1 ± 0.4	0.6 ± 0.1	2.0 ± 0.4	1.0 ± 0.2	2.9 ± 0.7*	1.9 ± 0.4**

*Note*: Hematocrit (%), Urinary sodium, chloride and potassium excretion levels before and during acute L‐NAME infusion (1.0 mg/kg/min, i.v) for 60 min in anesthetized male and female WKY. Data are expressed as mean ± SEM; *n* = 5–6 in each group.

* Indicates *p* < 0.05 versus baseline male or female WKY.

** Indicates *p* < 0.05 versus baseline female WKY only.

### 3.3 Selective NOS1 inhibition increases BP similarly in male and female WKY

**FIGURE 3:**
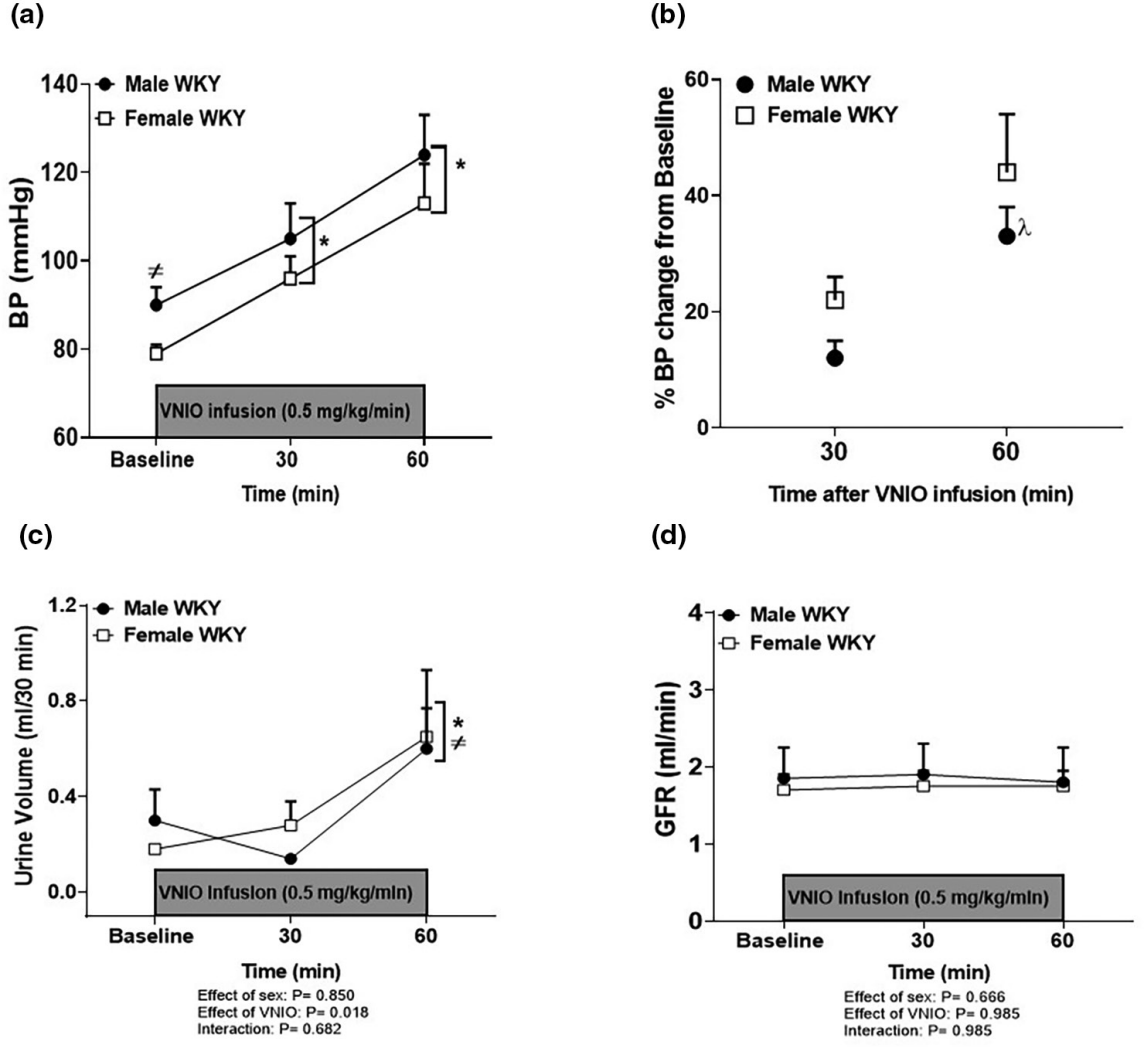
Selective NOS1 inhibition increases BP similarly in male and female WKY. BP prior to and during VNIO infusion (0.5 mg/kg/min, i.v) for 60 min in anesthetized male and female WKY (a). All data are expressed as mean ± SEM; *n* = 5–6 in each group. * Indicates *p* < 0.05 versus baseline values of either male or female WKY and ^≠^ indicates *p* < 0.05 versus baseline female WKY. Percent change in BP from baseline values in male and female WKY after 30 min and 60 min infusion of VNIO (b). ^λ^ Indicates *p* < 0.05 versus male WKY after 30 min of VNIO infusion. Urine volume (c) and glomerular filtration rate (GFR, d) prior to and during VNIO infusion (0.5 mg/kg/min, i.v) for 60 min in anesthetized male and female WKY. * Indicates *p* < 0.05 versus baseline male or female WKY and ^≠^ indicates *p* < 0.05 versus 30 infusion of VNIO in male or female WKY.

NOS1 has been linked to BP control and females have greater renal NOS1 expression than males (Sullivan et al., 2010). As a result, additional studies were designed to examine the relative contribution of NOS1 to the enhanced BP sensitivity of females to total NOS inhibition with L‐NAME. However, sex differences in BP were maintained throughout all doses of VNIO bolus injection (Figure 1b) and 60‐min infusion of VNIO at doses higher than 0.5 mg/kg/min resulted in ~70% mortality rates in both sexes. Therefore, 0.5 mg/kg/min was selected for additional studies. Selective NOS1 inhibition increased BP in both male and female WKY (Figure 3a), and the increases in BP were comparable between the sexes following both 30‐ and 60‐min VNIO infusion (Figure 3b).

Infusion of VNIO for 60 min also significantly increased urine volume comparably in male and female WKY (Figure 3c, effect of sex, *p* = 0.850; effect of VNIO, *p* = 0.018; interaction, *p* = 0.682). There were no changes in hematocrit (Table 2, effect of sex, *p* = 0.05; effect of VNIO, *p* = 0.731; interaction, *p* = 0.902) or GFR with VNIO in either sex (Figure 3d, effect of sex, *p* = 0.666; effect of VNIO, *p* = 0.985; interaction, *p* = 0.985). Urinary sodium and chloride excretion levels significantly increased in both sexes after VNIO infusion compared to baseline values, and there were no sex differences in excretion levels (Table 2). There was no significant change in urinary potassium excretion in either sex with VNIO infusion (Table 2).

**TABLE 2:** VNIO increased urinary sodium, chloride and potassium excretion levels compared to baseline values without changing hematocrit values.

Parameter	Baseline		30 min after VNIO infusion		60 min after VNIO infusion	
Hematocrit (%)	Male	Female	Male	Female	Male	Female
	44 ± 1.0	40.0 ± 2.0	43 ± 1.0	40 ± 1.0	44 ± 1.3	41.0 ± 1.0
Na+ Excretion (μmol/min)	Male	Female	Male	Female	Male	Female
	0.1 ± 0.01	0.04 ± 0.002	0.12 ± 0.02	0.12 ± 0.4	1.5 ± 0.6*	2.0 ± 0.7*
Cl− Excretion (μmol/min)	Male	Female	Male	Female	Male	Female
	0.44 ± 0.1	0.46 ± 0.15	1.3 ± 0.3	0.6 ± 0.2	3.3 ± 1.0*	3.1 ± 1.0*
K+ Excretion (μmol/min)	Male	Female	Male	Female	Male	Female
	1.2 ± 0.3	1.03 ± 0.5	1.6 ± 0.5	2.75 ± 1.0	2.8 ± 0.8	3.2 ± 1.0

*Note*: Hematocrit (%), urinary sodium, chloride and potassium excretion levels before and during acute VNIO infusion (0.5 mg/kg/min, i.v) for 60 min in anesthetized male and female WKY. Data are expressed as mean ± SEM; *n* = 5–6 in each group.

* Indicates *p* < 0.05 versus baseline male or female WKY.

## 4 DISCUSSION

The current study investigated the impact of biological sex on the sensitivity of BP and renal hemodynamic responses to acute NOS inhibition in male and female normotensive WKY. The main finding of the current study is that females are more sensitive to nonselective NOS inhibition‐induced increases in BP than males, supporting the notion that females are more dependent on NOS to control BP versus males under normotensive baseline conditions. Moreover, greater BP sensitivity in females was not due to enhanced renal hemodynamic responses or changes in electrolyte excretion. Our findings demonstrated no apparent sex differences in response to specific NOS1 inhibition, further suggesting that greater renal NOS3 activity or expression likely mediates the enhanced BP response in females.

Previous findings have indicated that both normotensive and hypertensive female rats have more NO than males (Brinson et al., 2013; Glushkovskaya‐Semyachkina et al., 2006; Loria et al., 2014; Sullivan et al., 2010; Wang et al., 2003). Despite this consistent finding, there are conflicting data in the literature regarding the impact of NOS inhibition on BP in normotensive rats (Sainz et al., 2004; Wang et al., 2003; Wu et al., 2001). Previous findings suggest that L‐NAME infusion at 50–200 μg/kg/min dose range increased BP in rats (Dobrowolski et al., 2015; Nafrialdi et al., 1994). However, giving the important role of the kidney in BP regulation, we initially identified a dose of L‐NAME that abolished the sex difference in BP in WKY before infusing this dose into both male and female WKY rats for 60 min to determine if sex differences in BP responses to NOS inhibition also coincide with sex differences in hemodynamic responses to NOS inhibition. Consistent with Wang et al. (Wang et al., 2003), our findings suggest that BP in normotensive females are more sensitive to nonspecific NOS inhibition with L‐NAME than males confirming the important role of NO in baseline BP regulation in normotensive, healthy rats. Due to the important roles played by NO in BP control, and the fact that there are numerous NOS isoforms, the mechanism which enhances BP sensitivity to NO in females relative to males is unknown.

The role of the kidney in BP regulation is well‐established and hypertension is often associated with renal hemodynamic changes (Munger & Baylis, 1988; Reckelhoff et al., 1998). In the kidney, NO is a key factor in regulating urine volume and sodium excretion via changing renal hemodynamics and/or salt and water handling by the nephron (Lee, 2008). NO dilates afferent arterioles, reduces renal vascular resistance, and increases GFR (Lee, 2008; Romero & Carretero, 2019). NO also regulates renin secretion, tubule‐glomerular feedback and transport in various nephron segments (Lee, 2008, Romero & Carretero, 2019). Inhibition of NOS significantly increases BP and renal vascular resistance leading to a fall in renal blood flow and small fall in GFR due to increased filtration fraction in WKY (Baylis & Qiu, 1996; Granstam et al., 1998; Reckelhoff et al., 1998). Sex differences in BP have been reported to influence renal function and the progression of renal disease (Coggins et al., 1998; Neugarten, 2002; Silbiger & Neugarten, 2003). In normotensive rats, chronic L‐NAME treatment increases BP and renal vascular resistance and decreases renal blood flow without significant changes in GFR in either sex and with no apparent sex differences (Reckelhoff et al., 1998). Although the elevation in BP following acute NOS inhibition with L‐NAME in the current study resulted in increased urine volume and sodium and chloride excretion levels in both sexes, GFR was not altered in either sex following 60 min of infusion. Furthermore, there were no apparent sex differences in renal hemodynamic responses to acute NOS inhibition infusion in WKY. Thus, the elevation in urine volume and electrolyte excretion can likely be attributed to the increase in BP and renal perfusion pressure following L‐NAME infusion.

NOS1 has been shown to play a role in BP regulation and renal hemodynamics (Sasser et al., 2015; Sullivan et al., 2010) and chronic treatment with a pharmacological NOS1 inhibitor significantly increases BP in male Wistar rats (Cacanyiova et al., 2009). In the kidney, efferent arteriole constriction to angiotensin II is exacerbated in the presence of a NOS1 inhibitor in male Sprague–Dawley rats (Ichihara et al., 1998), and genetic deletion of NOS1 decreases medullary blood flow in response to angiotensin II (Mattson & Meister, 2005) suggesting an important role of NOS1 in the regulation of renal hemodynamics. As renal NOS1 has been linked to BP control (Mattson & Bellehumeur, 1996), we further expand our study to determine whether NOS1 differentially contributes to BP regulation in male and female WKY. Previous findings found that l‐VNIO infused to achieve a concentration of 1 μmoL/L in renal arterial blood augmented myogenic autoregulation in male Wistar rats, yet this concentration did not cause significant vasoconstriction (Shi et al., 2006). However, addressing the effect of NOS1 inhibition with VNIO on BP acutely has not been explored. Our initial studies were unsuccessful in identifying a dose of VNIO that would abolish sex difference in BP in WKY suggesting that NOS1 might not drive the enhanced BP sensitivity of females versus male WKY. Moreover, VNIO injection at doses higher than 0.5 mg/kg increased mortality rates in both sexes suggesting WKY are highly sensitive to selective NOS1‐inhibition. We found no sex differences in BP or hemodynamic responses to NOS1 inhibition. These data both confirm the important role of NOS1 in BP regulation in WKY and suggest that NOS1 is not the isoform driving enhanced BP sensitivity in females versus male WKY. As kidneys express both NOS1 and NOS3 (Hyndman et al., 2013; Sasser et al., 2015), our findings suggest greater NOS3‐depedent control of BP in females.

While there are three NOS isoforms, the current study focused on NOS1 and NOS 3. NOS2 has not reliably been detected in the kidney of normotensive rats and we and others previously published no NOS2 activity or expression in the kidney of male or female SHR (Choi et al., 2012; Kosaka et al., 2003; Sullivan et al., 2010). Therefore, a role for NOS2 in BP regulation in normotensive rats is unlikely. NOS2 is expressed under pathological conditions such as following ischemia–reperfusion or lipopolysaccharide injections and could contribute to BP regulation during inflammatory condition (Choi et al., 2012; Kosaka et al., 2003; Sedaghat et al., 2019). Thus, the role of all NOS isoforms in BP regulation under physiological and pathophysiological condition is well‐established.

Our study have few limitations. Although our study addressed the effect of acute NOS inhibition on BP and renal hemodynamics in in both sexes of WKY rats, our design did not provide further mechanistic evidence. However, our findings suggest a mechanistic role for NOS3 as the primary isoform regulating BP in the normotensive females. Another limitation is the number of animal per group. Considering the L‐NAME treatment group reached significance, we are unsure as to what increasing the animal number per group will further accomplish. Finally, we notice difference between hematocrit values during L‐NAME acute infusion (Table 1) versus VNIO acute infusion (Table 2). Clinically, females have lower hematocrit values than males (female 35–45% vs. male 40–50%). As the infusion experiments for L‐NAME and VNIO were run in two different semesters (fall vs. spring), we could postulate seasonal change as a confounding factor for this discrepancy. However, although female WKY had lower hematocrit values versus male WKY during VNIO infusion consistent with the clinical scenario, these changes were not significant.

### 4.1 Perspectives and significance

Clinically, premenopausal females have lower BP than age‐matched males. Since NO bioavailability is greater in females than males and NO plays a key role in BP regulation, sex differences in NO bioavailability may be central to BP differences in males and females. Greater NO levels in females may offer renal vascular protection in females relative to males. Future studies will examine the mechanisms by which NOS is increased in females.

## ETHICS STATEMENT

All experiments were conducted in accordance with the National Institutes of Health (Guide for the Care and Use of Laboratory Animals) and approved and monitored by the Augusta University Institutional Animal Care and Use Committee as the institutional ethical committee. The institutional approval code/number is A3307‐01.We have taken all steps to minimize the animals’ pain and suffering during experimental procedures.

## References

[phy215771-bib-0001] Alhashim, A. , Abdelbary, M. , Sullivan, J. C. , Naeini, S. E. , & Elmarakby, A. A. (2022). Sexual dimorphism in renal heme oxygenase‐1 and arachidonic acid metabolizing enzymes in spontaneously hypertensive rats versus normotensive Wistar Kyoto rats. Prostaglandins & Other Lipid Mediators, 161, 106650. 35618157 10.1016/j.prostaglandins.2022.106650

[phy215771-bib-0002] Baylis, C. , & Qiu, C. (1996). Importance of nitric oxide in the control of renal hemodynamics. Kidney International, 49, 1727–1731. 8743486 10.1038/ki.1996.256

[phy215771-bib-0003] Brinson, K. N. , Elmarakby, A. A. , Tipton, A. J. , Crislip, G. R. , Yamamoto, T. , Baban, B. , & Sullivan, J. C. (2013). Female SHR have greater blood pressure sensitivity and renal T cell infiltration following chronic NOS inhibition than males. American Journal of Physiology. Regulatory, Integrative and Comparative Physiology, 305, R701–R710. 23883679 10.1152/ajpregu.00226.2013 PMC3798799

[phy215771-bib-0004] Cacanyiova, S. , Kristek, F. , Gerova, M. , Krenek, P. , & Klimas, J. (2009). Effect of chronic nNOS inhibition on blood pressure, vasoactivity, and arterial wall structure in Wistar rats. Nitric Oxide, 20, 304–310. 19303939 10.1016/j.niox.2009.03.002

[phy215771-bib-0005] Choi, J. Y. , Nam, S. A. , Jin, D. C. , Kim, J. , & Cha, J. H. (2012). Expression and cellular localization of inducible nitric oxide synthase in lipopolysaccharide‐treated rat kidneys. The Journal of Histochemistry and Cytochemistry, 60, 301–315. 22260992 10.1369/0022155411436131 PMC3351238

[phy215771-bib-0006] Coggins, C. H. , Breyer Lewis, J. , Caggiula, A. W. , Castaldo, L. S. , Klahr, S. , & Wang, S. R. (1998). Differences between women and men with chronic renal disease. Nephrology, Dialysis, Transplantation, 13, 1430–1437. 10.1093/ndt/13.6.1430 9641172

[phy215771-bib-0007] Del Campo, L. , Ferrer, M. , & Balfagon, G. (2009). Hypertension alters the function of nitrergic and sensory innervation in mesenteric arteries from female rats. Journal of Hypertension, 27, 791–799. 19516178 10.1097/HJH.0b013e32832531e6

[phy215771-bib-0008] Dobrowolski, L. , Kuczeriszka, M. , Castillo, A. , Majid, D. S. , & Navar, L. G. (2015). Role of atrial natriuretic peptide in mediating the blood pressure‐independent natriuresis elicited by systemic inhibition of nitric oxide. Pflügers Archiv, 467, 833–841. 24953240 10.1007/s00424-014-1557-4 PMC4276550

[phy215771-bib-0009] Elmarakby, A. A. , Bhatia, K. , Crislip, R. , & Sullivan, J. C. (2016). Hemodynamic responses to acute angiotensin II infusion are exacerbated in male versus female spontaneously hypertensive rats. Physiological Reports, 4, e12677. 26755738 10.14814/phy2.12677 PMC4760407

[phy215771-bib-0010] Glushkovskaya‐Semyachkina, O. V. , Anishchenko, T. G. , Sindyakova, T. A. , Leksina, O. V. , & Berdnikova, V. A. (2006). Sex‐related differences in nitric oxide content in healthy and hypertensive rats at rest and under stress conditions. Bulletin of Experimental Biology and Medicine, 142, 9–11. 17369889 10.1007/s10517-006-0277-y

[phy215771-bib-0011] Granstam, S. O. , Lind, L. , Granstam, E. , & Fellstrom, B. (1998). Effects of nitric oxide synthase inhibition and endothelin ETA receptor blockade on haemodynamics in hypertensive rats. Clinical and Experimental Pharmacology & Physiology, 25, 693–701. 9750958 10.1111/j.1440-1681.1998.tb02278.x

[phy215771-bib-0012] Herrera, M. , & Garvin, J. L. (2005). Recent advances in the regulation of nitric oxide in the kidney. Hypertension, 45, 1062–1067. 15753231 10.1161/01.HYP.0000159760.88697.1e

[phy215771-bib-0013] Hilliard, L. M. , Sampson, A. K. , Brown, R. D. , & Denton, K. M. (2013). The "his and hers" of the renin‐angiotensin system. Current Hypertension Reports, 15, 71–79. 23180053 10.1007/s11906-012-0319-y

[phy215771-bib-0014] Hyndman, K. A. , Boesen, E. I. , Elmarakby, A. A. , Brands, M. W. , Huang, P. , Kohan, D. E. , Pollock, D. M. , & Pollock, J. S. (2013). Renal collecting duct NOS1 maintains fluid‐electrolyte homeostasis and blood pressure. Hypertension, 62, 91–98. 23608660 10.1161/HYPERTENSIONAHA.113.01291 PMC3901402

[phy215771-bib-0015] Ichihara, A. , Inscho, E. W. , Imig, J. D. , & Navar, L. G. (1998). Neuronal nitric oxide synthase modulates rat renal microvascular function. The American Journal of Physiology, 274, F516–F524. 9530268 10.1152/ajprenal.1998.274.3.F516

[phy215771-bib-0016] Ide, T. , Tsutsui, H. , Ohashi, N. , Hayashidani, S. , Suematsu, N. , Tsuchihashi, M. , Tamai, H. , & Takeshita, A. (2002). Greater oxidative stress in healthy young men compared with premenopausal women. Arteriosclerosis, Thrombosis, and Vascular Biology, 22, 438–442. 11884287 10.1161/hq0302.104515

[phy215771-bib-0017] Kahonen, M. , Tolvanen, J. P. , Sallinen, K. , Wu, X. , & Porsti, I. (1998). Influence of gender on control of arterial tone in experimental hypertension. The American Journal of Physiology, 275, H15–H22. 9688891 10.1152/ajpheart.1998.275.1.H15

[phy215771-bib-0018] Kosaka, H. , Yoneyama, H. , Zhang, L. , Fujii, S. , Yamamoto, A. , & Igarashi, J. (2003). Induction of LOX‐1 and iNOS expressions by ischemia‐reperfusion of rat kidney and the opposing effect of L‐arginine. The FASEB Journal, 17, 636–643. 12665476 10.1096/fj.02-0585com

[phy215771-bib-0019] Layton, A. T. , & Sullivan, J. C. (2019). Recent advances in sex differences in kidney function. American Journal of Physiology. Renal Physiology, 316, F328–F331. 30565997 10.1152/ajprenal.00584.2018 PMC6397367

[phy215771-bib-0020] Lee, J. (2008). Nitric oxide in the kidney: Its physiological role and pathophysiological implications. Electrolyte Blood Press, 6, 27–34. 24459519 10.5049/EBP.2008.6.1.27 PMC3894485

[phy215771-bib-0021] Li, Q. , Youn, J. Y. , & Cai, H. (2015). Mechanisms and consequences of endothelial nitric oxide synthase dysfunction in hypertension. Journal of Hypertension, 33, 1128–1136. 25882860 10.1097/HJH.0000000000000587 PMC4816601

[phy215771-bib-0022] Loria, A. S. , Brinson, K. N. , Fox, B. M. , & Sullivan, J. C. (2014). Sex‐specific alterations in NOS regulation of vascular function in aorta and mesenteric arteries from spontaneously hypertensive rats compared to Wistar Kyoto rats. Physiological Reports, 2, e12125. 25168874 10.14814/phy2.12125 PMC4246578

[phy215771-bib-0023] Mattson, D. L. , & Bellehumeur, T. G. (1996). Neural nitric oxide synthase in the renal medulla and blood pressure regulation. Hypertension, 28, 297–303. 8707397 10.1161/01.hyp.28.2.297

[phy215771-bib-0024] Mattson, D. L. , & Meister, C. J. (2005). Renal cortical and medullary blood flow responses to L‐NAME and ANG II in wild‐type, nNOS null mutant, and eNOS null mutant mice. American Journal of Physiology. Regulatory, Integrative and Comparative Physiology, 289, R991–R997. 15961532 10.1152/ajpregu.00207.2005

[phy215771-bib-0025] Mcintyre, M. , Hamilton, C. A. , Rees, D. D. , Reid, J. L. , & Dominiczak, A. F. (1997). Sex differences in the abundance of endothelial nitric oxide in a model of genetic hypertension. Hypertension, 30, 1517–1524. 9403576 10.1161/01.hyp.30.6.1517

[phy215771-bib-0026] Munger, K. , & Baylis, C. (1988). Sex differences in renal hemodynamics in rats. The American Journal of Physiology, 254, F223–F231. 3344806 10.1152/ajprenal.1988.254.2.F223

[phy215771-bib-0027] Nafrialdi, N. , Jover, B. , & Mimran, A. (1994). Endogenous vasoactive systems and the pressor effect of acute N omega‐nitro‐L‐arginine methyl ester administration. Journal of Cardiovascular Pharmacology, 23, 765–771. 7521459 10.1097/00005344-199405000-00011

[phy215771-bib-0028] Neugarten, J. (2002). Gender and the progression of renal disease. Journal of the American Society of Nephrology, 13, 2807–2809. 12397053 10.1097/01.asn.0000035846.89753.d4

[phy215771-bib-0029] Reckelhoff, J. F. , Hennington, B. S. , Moore, A. G. , Blanchard, E. J. , & Cameron, J. (1998). Gender differences in the renal nitric oxide (NO) system: Dissociation between expression of endothelial NO synthase and renal hemodynamic response to NO synthase inhibition. American Journal of Hypertension, 11, 97–104. 9504456 10.1016/s0895-7061(97)00360-9

[phy215771-bib-0030] Romero, C. A. , & Carretero, O. A. (2019). Tubule‐vascular feedback in renal autoregulation. American Journal of Physiology. Renal Physiology, 316, F1218–F1226. 30838873 10.1152/ajprenal.00381.2018 PMC6620598

[phy215771-bib-0031] Sabolic, I. , Asif, A. R. , Budach, W. E. , Wanke, C. , Bahn, A. , & Burckhardt, G. (2007). Gender differences in kidney function. Pflügers Archiv, 455, 397–429. 17638010 10.1007/s00424-007-0308-1

[phy215771-bib-0032] Sainz, J. , Osuna, A. , Wangensteen, R. , De Dios Luna, J. , Rodriguez‐Gomez, I. , Duarte, J. , Moreno, J. M. , & Vargas, F. (2004). Role of sex, gonadectomy and sex hormones in the development of nitric oxide inhibition‐induced hypertension. Experimental Physiology, 89, 155–162. 15123544 10.1113/expphysiol.2003.002652

[phy215771-bib-0033] Sandberg, K. , & Ji, H. (2012). Sex differences in primary hypertension. Biology of Sex Differences, 3, 7. 22417477 10.1186/2042-6410-3-7 PMC3331829

[phy215771-bib-0034] Sasser, J. M. , Brinson, K. N. , Tipton, A. J. , Crislip, G. R. , & Sullivan, J. C. (2015). Blood pressure, sex, and female sex hormones influence renal inner medullary nitric oxide synthase activity and expression in spontaneously hypertensive rats. Journal of the American Heart Association, 4, 1–8. 10.1161/JAHA.114.001738 PMC4579936 25862792

[phy215771-bib-0035] Sedaghat, Z. , Kadkhodaee, M. , Seifi, B. , & Salehi, E. (2019). Inducible and endothelial nitric oxide synthase distribution and expression with hind limb per‐conditioning of the rat kidney. Archives of Medical Science, 15, 1081–1091. 31360203 10.5114/aoms.2019.85651 PMC6657261

[phy215771-bib-0036] Shi, Y. , Wang, X. , Chon, K. H. , & Cupples, W. A. (2006). Tubuloglomerular feedback‐dependent modulation of renal myogenic autoregulation by nitric oxide. American Journal of Physiology. Regulatory, Integrative and Comparative Physiology, 290, R982–R991. 16293681 10.1152/ajpregu.00346.2005

[phy215771-bib-0037] Silbiger, S. R. , & Neugarten, J. (2003). The role of gender in the progression of renal disease. Advances in Renal Replacement Therapy, 10, 3–14. 12616458 10.1053/jarr.2003.50001

[phy215771-bib-0038] Stuehr, D. J. (2004). Enzymes of the L‐arginine to nitric oxide pathway. The Journal of Nutrition, 134, 2748S–2751S. 15465779 10.1093/jn/134.10.2748S

[phy215771-bib-0039] Sullivan, J. C. , Pardieck, J. L. , Hyndman, K. A. , & Pollock, J. S. (2010). Renal NOS activity, expression, and localization in male and female spontaneously hypertensive rats. American Journal of Physiology. Regulatory, Integrative and Comparative Physiology, 298, R61–R69. 19889864 10.1152/ajpregu.00526.2009 PMC2806203

[phy215771-bib-0040] Villanueva, C. , & Giulivi, C. (2010). Subcellular and cellular locations of nitric oxide synthase isoforms as determinants of health and disease. Free Radical Biology & Medicine, 49, 307–316. 20388537 10.1016/j.freeradbiomed.2010.04.004 PMC2900489

[phy215771-bib-0041] Wang, Y. R. , Yen, C. H. , Sun, Y. F. , & Laun, Y. T. (2003). Gender‐dependent response in blood pressure changes following the inhibition of nitric oxide synthase. The Chinese Journal of Physiology, 46, 91–94. 12974300

[phy215771-bib-0042] Wiinberg, N. , Hoegholm, A. , Christensen, H. R. , Bang, L. E. , Mikkelsen, K. L. , Nielsen, P. E. , Svendsen, T. L. , Kampmann, J. P. , Madsen, N. H. , & Bentzon, M. W. (1995). 24‐h ambulatory blood pressure in 352 normal Danish subjects, related to age and gender. American Journal of Hypertension, 8, 978–986. 8845079 10.1016/0895-7061(95)00216-2

[phy215771-bib-0043] Wu, Y. , Huang, A. , Sun, D. , Falck, J. R. , Koller, A. , & Kaley, G. (2001). Gender‐specific compensation for the lack of NO in the mediation of flow‐induced arteriolar dilation. American Journal of Physiology: Heart and Circulatory Physiology, 280, H2456–H2461. 11356598 10.1152/ajpheart.2001.280.6.H2456

